# Strawberry Tree Fruit Residue as Carbon Source Towards Sustainable Fuel Biodesulfurization by *Gordonia alkanivorans* Strain 1B

**DOI:** 10.3390/molecules30102137

**Published:** 2025-05-13

**Authors:** Susana M. Paixão, Tiago P. Silva, Francisco Salgado, Luís Alves

**Affiliations:** 1 Unidade de Bioenergia e Biorrefinarias, LNEG—Laboratório Nacional de Energia e Geologia, Estrada do Paço do Lumiar 22, 1649-038 Lisbon, Portugal; 2 Departamento de Alterações Climáticas, APA—Agência Portuguesa do Ambiente, Rua da Murgueira 9, 2610-124 Amadora, Portugal

**Keywords:** *Gordonia alkanivorans* strain 1B, strawberry tree fruit residue, biodesulfurization, bioproducts, carotenoids, gordofactin

## Abstract

Biodesulfurization (BDS) is a clean technology that uses microorganisms to efficiently remove sulfur from recalcitrant organosulfur compounds present in fuels (fossil fuels or new-generation fuels resulting from pyrolysis and hydrothermal liquefaction). One of the limitations of this technology is the low desulfurization rates. These result in the need for greater amounts of biocatalyst and lead to increased production costs. To mitigate this issue, several approaches have been pursued, such as the use of alternative carbon sources (C-sources) from agro-industrial waste streams or the co-production of high-added-value products by microorganisms. The main goal of this work is to assess the potential of strawberry tree fruit residue (STFr) as an alternative C-source for a BDS biorefinery using *Gordonia alkanivorans* strain 1B, a well-known desulfurizing bacterium with high biotechnological potential. Hence, the first step was to produce sugar-rich liquor from the STFr and employ it in shake-flask assays to evaluate the influence of different pretreatments (treatments with 1–4% activated charcoal for prior phenolics removal) on metabolic parameters and BDS rates. Afterwards, the liquor was used as the C-source in chemostat assays, compared to commercial sugars, to develop and optimize the use of STFr-liquor as a viable C-source towards cost-effective biocatalyst production. Moreover, the high-market-value bioproducts simultaneously produced during microbial growth were also evaluated. In this context, the best results, considering both the production of biocatalysts with BDS activity and simultaneous bioproduct production (carotenoids and gordofactin biosurfactant/bioemulsifier) were achieved when strain 1B was cultivated in a chemostat with untreated STFr-liquor (5.4 g/L fructose + glucose, 6:4 ratio) as the C-source and in a sulfur-free mineral-minimized culture medium at a dilution rate of 0.04 h^−1^. Cells from this steady-state culture (STFr L1) achieved the highest desulfurization with 250 mM of dibenzothiophene as a reference organosulfur compound, producing a maximum of ≈213 mM of 2-hydroxibyphenil (2-HBP) with a corresponding specific rate (*q*_2-HBP_) of 6.50 µmol/g(_DCW_)/h (where DCW = dry cell weight). This demonstrates the potential of STFr as a sustainable alternative C-source for the production of cost-effective biocatalysts without compromising BDS ability. Additionally, cells grown in STFr L1 also presented the highest production of added-value products (338 ± 15 µg/g_(DCW)_ of carotenoids and 8 U/mL of gordofactin). These results open prospects for a future *G. alkanivorans* strain 1B biorefinery that integrates BDS, waste valorization, and the production of added-value products, contributing to the global economic viability of a BDS process and making BDS scale-up a reality in the near future.

## 1. Introduction

Biodesulfurization (BDS) is a technique developed to remove sulfur from fuels at low temperatures and pressures to minimize energy spending and increase process efficiency. Instead of employing metal catalysts and molecular hydrogen, which are typically used in hydrodesulfurization, BDS takes advantage of the metabolic capabilities of microorganisms and acts directly on the sulfur atoms contained in the organosulfur compounds without lowering the final fuel quality [1]. BDS can be especially important when dealing with new-generation fuels resulting from the thermochemical processing of biomass (i.e., pyrolysis), since these can contain complex sulfur compounds that can be difficult to treat through conventional processes, but can be desulfurized through BDS [2,3]. Despite having clear industrial potential, this technique has yet to be successfully applied at an industrial level [4]. This is mostly due to the high costs of producing microorganisms (the biocatalysts), as well as the low BDS rates, which make the process too expensive. To solve this issue, it is necessary to reduce production costs and increase revenue sources. One strategy followed in other biotechnological processes [5] is to select microorganisms that can simultaneously desulfurize at significant rates and produce high-added-value products.

*G. alkanivorans* strain 1B [6] is a well-known bacterium with high biotechnological potential. In the last decade, several works have shown its great BDS ability, permitting the effective desulfurization of several recalcitrant organosulfur compounds, fossil fuels, and new-generation fuels (e.g., pyrolysis fuels, bio-oils) [7,8,9,10,11]. Moreover, it was also demonstrated that this bacterium can produce high-added-value products, such as carotenoids [12,13,14,15] and biosurfactants/bioemulsifiers (BS/BE) [16,17,18], and it can grow on vast C-sources [19,20,21,22,23].

Another strategy often selected for cost reduction is the use of alternative, cheap, and renewable C-sources [24,25,26]. Due to their low cost, the utilization of agro-industrial residues or other waste materials is one of the most followed strategies in biotechnological processes using heterotrophic cultures [9,21,27]. Considering strain 1B’s fructophilic behavior [28], fructose-rich alternative C-sources will be preferential choices to produce highly effective BDS biocatalysts. However, it is necessary to account for their associated problems, such as the inherent toxicity of their components [19,22,29,30]. On the other hand, their influence on the concurrent production of high-added-value products must also be assessed, since, as mentioned above, these can then be further valued through other production cycles [9,13,31].

The strawberry tree, *Arbutus unedo* L., is an evergreen shrub belonging to the genus *Arbutus* of the *Ericaceae* family. It grows to 9–12 m tall but is typically between 1.5 m and 3 m tall [32]. Widespread in the Mediterranean Basin and the Atlantic coast of Western Europe [33,34], with high tolerance to dryness, *A. unedo* occurs in areas with poor siliceous soils and high slopes [35], being one of the most important species of the Mediterranean maquis and Iberian heaths. In Portugal, it occurs throughout the country, but with a greater concentration in the Algarve region (Caldeirão and Monchique mountains). As a naturally occurring plant, it is fundamental to maintain local ecosystems, but it also has other important characteristics: it regenerates rapidly after fires, which is especially important in southern Europe, where fires are a repeat occurrence; it grows on poor soils, which contributes to preventing erosion; and it can be grown on contaminated soils as a form of phytoremediation, namely against arsenic contamination [33]. Moreover, it is abundantly used in Mediterranean folk medicine, likely due to the high content of polyphenols and other phytochemicals [30,36,37].

Strawberry tree fruits (STFs) ripen from late September/mid-October to early December; they are globular berries, up to 2–3 cm in diameter with a rough surface, and are green or orange to red in color. In Portugal, one of the main STF producers, it was estimated that production increased from 500 to 700 tons/year in 2019 [38] to >6000 tons/year in 2022 [39]. These fruits are rich in sugars (42–52%, dry-weight basis, mostly fructose and glucose), omega-3 polyunsaturated fatty acids, minerals, vitamins, organic acids, and phenolic compounds [40]. Despite its characteristics, STF is rarely consumed as a fresh fruit, typically being processed into different products, the most famous of which is a distilled beverage known as “Aguardente de medronho” in Portugal, “Koumaro” in Greece, and “Corbezzolo” in Italy [40], constituting an agro-sustainable business. STF is also frequently used to produce jams or jellies and marmalades, resulting in the production of sugar-rich residues [41].

In this context, the main goal of this study was the valorization of STF residue (STFr), mimicking that resulting from the manufacturing process of jams, jellies, and marmalades, as a low-cost feedstock for a sustainable BDS refinery using *Gordonia alkanivorans* strain 1B as the desulfurizing microorganism. Hence, fructose-rich liquors obtained from STFr were evaluated as an alternative C-source for coupled dibenzothiophene (DBT) desulfurization and bioproduct (carotenoids, gordofactin) production, aiming to achieve a sustainable bioprocess scale-up.

## 2. Materials and Methods

### 2.1. Chemicals

Dibenzothiophene (DBT 99%) was obtained from Acros Organics (Geel, Belgium), 2-hydroxybiphenyl (2-HBP), dimethylformamide (DMF) and 4-methyl dibenzothiophene (4-mDBT 96%) were from Sigma-Aldrich (Saint Louis, MO, USA). A DBT stock solution (150 mM) was prepared by dissolving it in dimethylformamide (DMF) to facilitate manipulation, preservation, and reproducibility. Sodium sulfate anhydrous (>99%) and activated charcoal were obtained from Merck, and polypropylene glycol (PPG-2000) was obtained from BDH Chemicals (Radnor, PA, USA). All other reagents were of the highest grade commercially available. Stock solutions of sodium sulfate (20 g/L) and fructose (fru)/glucose (glu) mix (50% *w*/*v*) were previously prepared and filter-sterilized (0.20 µm cellulose nitrate filters) before further use as sulfur/carbon sources (S/C-sources), respectively. Ultrapure water from Purist Ultrapure Water Systems (18.2 MΩ, PURIST^®^, RephiLe Bioscience Ltd., Lisbon, Portugal) was used for all experiments. Tributyl(ethyl)phosphonium diethyl phosphate ionic liquid from Solchemar (Alcácer do Sal, Portugal) and ethyl acetate (99.8%) from Carlo Erba (Emmendingen, Germany) were used for carotenoid extraction.

### 2.2. Strawberry Tree Fruit Residue Liquor Preparation

Strawberry tree fruits (STFs) were harvested in Oleiros, Castelo Branco, Portugal. Each batch of STF collected was processed to separate the pulp from the STF residue (STFr), which is mostly composed of peels and seeds, to replicate the residue obtained from a jam manufacturing plant. The residue was weighed and mixed with an equal part of ultrapure water and then mixed by inversion for 24 h at 4 °C to minimize contamination and consequently sugar loss and nutrient degradation. The resulting mix was centrifuged for 20 min at 10,000× *g* at 4 °C (Sigma 6-16KS, rotor 12600, Darmstadt, Germany) to collect the extract (liquor), which was frozen until needed (Figure S1 in Supplementary Material). This liquor was defrosted in water at room temperature, and then successively filtered through 8 µm filter paper, a 1.2 µm glass fiber filter, a 0.45 µm cellulose nitrate filter, and lastly a sterile 0.22 µm nitrocellulose filter in a filtration system and kept at 4 °C until further use.

#### Detoxification

To detoxify the STFr-liquor, 150 mL was placed in 500 mL shake flasks with 1, 2, 3, 4 and 5% (*w*/*v*) activated charcoal in an incubator (Unitron CH-4103, Infors AG, Bottingen, Switzerland) at 25 °C and 100 rpm. After 1 h, the detoxified SFTr liquors were collected and centrifuged (Sigma model 6-16KS, rotor 12600, Germany) for 30 min at 13,000× *g* to remove the charcoal. Subsequently, the liquors were subjected to sterilizing filtration as described above.

### 2.3. Microorganism and Culture Conditions

*Gordonia alkanivorans* strain 1B was the bacterium used for all assays in this study [6]. The medium used for the cultivation/maintenance of this microorganism and in the shake-flask assays was sulfur-free mineral (SFM) culture medium [28] at a pH of 7.5 ± 0.2.

In the chemostat assays, different culture medium formulations were tested: (1) the SFM culture medium as described above for 10 g/L C-source; (2) SFM-minimized (SFMM) culture medium: 1.098 g/L NH_4_Cl, 0.50 g/L KH_2_PO_4_, 0.50 g/L Na_2_HPO_4_.2H_2_O, 0.0425 g/L MgCl_2_.6H_2_O, 0.03254 g/L Na_2_SO_4_, ≈10 g/L C-source, and 0.125 mL/L of TES, with a final pH of 7.5 [8]; (3) SFM culture medium at half nutrient composition and 5 g/L C-source; and (4) SFM-minimized culture medium at half nutrient composition and 5 g/L C-source. In addition, polypropylene glycol was added to the culture medium in all assays at a concentration of 0.15 mL/L to control foam formation. Culture media were sterilized by autoclaving at 121 °C and 1 atm for 25 min.

The inoculum was prepared by cultivating strain 1B in a 500 mL shake with 100 mL of SFM medium with fructose (5 g/L) as a single C-source and 150 µM DBT as a single S-source for 72 h in a horizontal incubator (Unitron CH-4103, Infors AG, Bottingen, Switzerland) at 30 °C and 150 rpm of agitation.

### 2.4. BDS Assays with Growing Cells

BDS growing tests were performed in 500 mL shake flasks with 100 mL of culture medium containing STFr-liquor, with an initial concentration of ≈14 g/L of total reducing sugars (fructose + glucose), 400 μM of DBT, and 2–4% (*v*/*v*) of inoculum. Flasks were placed in an orbital incubator at 30 °C and 150 rpm of agitation in triplicate, and the assays were carried out until no growth was observed.

### 2.5. Chemostat Assays

The chemostat assays were performed on a BioFlo IIc bioreactor (New Brunswick Scientific, NJ, USA) with a 1 L vase with 435 mL working volume. Volume was maintained at a constant level through a surface dipped leveling tube connected to a peristaltic pump. In the chemostat, the temperature was kept at 30 °C, the pH was controlled by a pH probe and maintained at 7.5 by the addition of 1 M NaOH solution on demand, and agitation was kept at 410 rpm. The aeration rate was 2 vvm (vol. of air/vol. liquid/minute), safeguarded by 3 air filters, with pores of 0.22 µm and 1 µm, to prevent contamination. An O_2_ electrode was used to measure the dissolved oxygen and a gas analyzer (Servomex 1440 (O_2_/CO_2_), Sussex, UK) was used to measure the O_2_ and CO_2_ in the gas. Sampling was performed under aseptic conditions.

Three different C-sources were tested: (i) STFr-L: untreated STFr-liquor; (ii) DTX STFr-L: STFr-liquor detoxified with activated carbon; and (iii) Fru + Glu: a mixture of commercial fructose and glucose (6:4 ratio of fru–gluc, respectively, to mimic the STFr-liquor). The Fru + Glu solution was prepared by weighing both sugars individually and diluting them in ultrapure water up to 10% of the total media volume. Afterwards, this solution was filter-sterilized through filtration using a 0.22 μm pore sterile cellulose nitrate filter, before being added to the remaining culture medium. For all assays, the filter-sterilized C-sources were mixed with the remaining culture medium in a laminar flow chamber to prevent contamination. Furthermore, initial concentrations of the C-sources of about 5–10 g/L total sugars were tested.

In the bioreactor, an inoculum of 10% (*v*/*v*) was used to start the culture and the continuous feed was initiated within 24 h of inoculation. After 5 turnovers of culture medium inside the vase, the cultures were considered at a steady state (SS), i.e., when the growth occurs at a constant rate and all the culture parameters remain stable. Several parameters were monitored, such as dilution rate, temperature, pH, aeration, agitation, bioproduct concentration, residual reducing sugars (fructose/glucose, g/L), and cell density, with biomass concentration expressed as dry cell weight (DCW, g/L) and optical density at λ = 600 nm (OD_600_).

Based on data collected for each #SS-culture, several metabolic parameters (e.g., substrate consumption, carbon recovery, respiratory quotient, specific rate of C-source/S-source consumption, specific rate of CO_2_ production, specific rate of O_2_ utilization, carbon conversion efficiency) were calculated according to the equations described by Roseiro et al. [42] and Silva et al. [10].

### 2.6. BDS Assays Using Resting Cells

To evaluate the BDS ability of the cells produced in the chemostat assays, cells were collected during each steady state (#SS), washed, and prepared as previously described by Silva et al. [10]. The final samples, i.e., cells resuspended in phosphate solution (solution of 2.55 g/L KH_2_PO_4_ and 2.55 g/L Na_2_HPO_4_^.^2H_2_O, pH 7.5), contained about 7–10 g_(DCW)_/L (i.e., resting cells (RC) suspension). Then, the corresponding BDS assays were performed in 100 mL shake flasks with 25 mL of RC and 250 μM of DBT, at 30 °C and 150 rpm. These BDS assays were performed in triplicate and sampling was performed every 30 min (or 1 h) for at least four hours.

### 2.7. Screening of Added-Value Bioproducts

#### 2.7.1. Carotenoid Extraction from Biomass

The carotenoids produced by strain 1B in the best chemostat cultures (#SS-cultures) were evaluated according to the methodology recently developed by Silva et al. (2024) [43]. For each #SS-culture, pink colored biomass was collected on ice and concentrated through centrifugation to obtain a cell density of about 36 g_(DCW)_/L. Then, 400 µL of wet biomass was mixed with 50 µL of tributyl(ethyl)phosphonium diethyl phosphate ionic liquid and 1125 µL of ethyl acetate (co-solvent) in a 15 mL tube and further processed as described by Silva et al. [43]. Cells were extracted 3 times to achieve maximum carotenoid yield. Extraction assays were performed in triplicate and results were evaluated in terms of total carotenoid extracted (µg/g_(DCW)_), as described in Section 2.8.4.

#### 2.7.2. Biosurfactant/Bioemulsifer Compounds in Culture Broth

The #SS-cultures were also sampled to evaluate the presence of gordofactin, the biosurfactant/bioemulsifer (BS/BE) compound characteristic of *G. alkanivorans* strain 1B [18], in culture broth. Hence, cells were collected from each #SS-culture and further centrifuged (8600× *g* at 4 °C, 20 min) to recover the respective supernatants. Then, the emulsifying activity in these supernatants was quantified for gordofactin screening (Section 2.8.5).

### 2.8. Analytical Methods

#### 2.8.1. Optical Density and Dry Cell Weight

The culture growth was monitored by the analysis of optical density at λ = 600 nm (OD_600_) in a Thermo Scientific Genesys 20 spectrophotometer (Thermo Fisher Scientific Inc., Waltham, MA, USA), and dry cell weight was evaluated (DCW). DCW was determined by centrifuging 1.5 mL samples of the bacterial culture broth in previously weighted microcentrifuge tubes for 10 min at 15,000× *g* at room temperature in a Biofuge 15 centrifuge (Heraeus Sepatech, Hanau, Germany) and drying them overnight at 100 °C.

#### 2.8.2. DBT Desulfurization Evaluation

Desulfurization was evaluated by measuring 2-HBP production (2-HBP results from the desulfurization of DBT through the 4S-pathway). The samples (0.750 mL) were acidified with 25 µL of HCl (4 M) and extracted with 1.5 mL of ethyl acetate on a vortex (5 min) to extract 2-HBP and DBT. After phase separation, the organic phase (upper phase) was collected and 2-HBP concentration was quantified by high-performance liquid chromatography (HPLC) using an Agilent 1100 Series system (Agilent Technologies, Waldbronn, Germany) equipped with a diode array detector (DAD), reading at 220 nm, and an Agilent Eclipse PAH (5 µm, 4.6 × 250 mm) column. The mobile phase was a mix of acetonitrile and water at a flow rate of 0.420 mL/min. Samples ran for 23 min according to the gradient 0.9 min at 60% water. From 0.9 to 12 min water was gradually reduced to 0%, from 12 to 14.50 min 0% water was maintained, from 14.50 to 22 min the water was gradually increased to 60%, and from 22 to 23 min water was kept at 60%.

#### 2.8.3. Sugar Consumption Evaluation

Glucose and fructose concentrations were measured by HPLC instrumentation (LabChrom Merck/Hitachi), equipped with a differential refractive index detector and a Waters SugarPak 1 column (6.5 × 300 mm, Waters Corporation, Milford, MA, USA), as described by Fernandes et al. [13]. For sugar quantification, the samples of culture broth were prepared by centrifuging for 10 min at 15,000× *g* (Biofuge 15 centrifuge, Heraeus Sepatech, Hanau, Germany) at room temperature to collect the supernatant.

#### 2.8.4. Carotenoid Analysis

Carotenoid concentration was evaluated through spectroscopy analysis. To assess the amount of total carotenoids extracted from each biomass sample (µg/g_(DCW)_), the UV–Visible spectrum was run on a Shimadzu spectrophotometer UV-2401PC (Shimadzu Corporation, Kyoto, Japan) between 380 and 900 nm. There were peaks at around λ = 477 nm due to the carotenoid range, and the concentration of total carotenoids was estimated based on the Lambert–Beer equation according to Nobre et al. [44], but using the value of 2091.4 L/10 g/cm for the specific optical extinction coefficient at λ = 477 nm (wavelength of the maximum absorbance of canthaxanthin in ethyl acetate) [12,44].

#### 2.8.5. Gordofactin Screening: Emulsifying Activity Quantification

Gordofactin production ability by *G. alkanivorans* strain 1B grown in the chemostat was evaluated by determining the emulsifying activity (EA) in each #SS-supernatant. The EA was determined according to the new methodology developed by Tavares et al. [16].

Using 4 mL screw-cap glass tubes (10 × 75 mm, ND10 caps with PTFE septum), Tavares et al. [16] proposed assessing the emulsifying activity of a product by adding 1 mL of n-heptane to 1 mL of an aqueous solution containing the test product (e.g., supernatant), mixing by vortexing at high speed (2 min), and allowing the mixture to rest for 10 min before analyzing. So, for each #SS-supernatant, a set of emulsification tests with increasing volumes of supernatant (up to 1 mL) were carried out until 100% emulsion was obtained in the organic phase.

According to Tavares et al. [16], one emulsification unit was defined as the minimum volume of product (Vol_min_ of emulsifier/surfactant, up to 1 mL) needed to form and maintain 100% emulsion in the organic phase. Hence, the corresponding emulsifying activity (EA) value in U/mL was calculated as follows: [EA (product) = 1 U/Vol_min_ (mL)].

#### 2.8.6. Total Phenolic Quantification

Total phenolic content in the STFr-liquors of the different batches (lot #1 and #2) was measured by the Folin–Ciocalteu method [45] before and after the detoxification procedure.

## 3. Results and Discussion

### 3.1. Production of Sugar-Rich Liquor

One way to lower biocatalyst production costs and maximize the economic viability of the BDS process using *G. alkanivorans* strain 1B is to use alternative C-sources based on residues or sustainable sources rich in fructose. With this goal, the first step of this work was to produce a sugar-rich liquor from STFr. So, in the first lot, 2700 g of STF was weighed, mashed, and sieved, separating the pulp (1232 g) and residue (mostly seeds and peels (1318 g)), mimicking the process at a jam manufacture facility. Then, STFr was extracted with water to produce a sugar-rich liquor, as schematized in Figure S1 (see Section 2.2).

Three lots of STFr-liquor were prepared during this study. Their characterization in terms of sugars (fru/gluc) and total phenolics is summarized in Table 1. The different lots obtained demonstrate the variability that results from working with an agro-industrial residue. Lot #1 and lot #3 were very similar, even though they were prepared with residues from fruits of different trees obtained in different years, while lot #2 presents a much lower sugar concentration. This discrepancy could be the result of the fruits being at different maturation stages, or differences in the manufacturing process of the jam. However, it is important to indicate that the sugar proportion (i.e., fru–gluc ratio) was maintained (6:4) in all liquors, despite the different total sugar concentrations.

### 3.2. Biodesulfurization Assays

#### 3.2.1. Preliminary Growth Assays with STFr-Liquor

**Figure 1:**
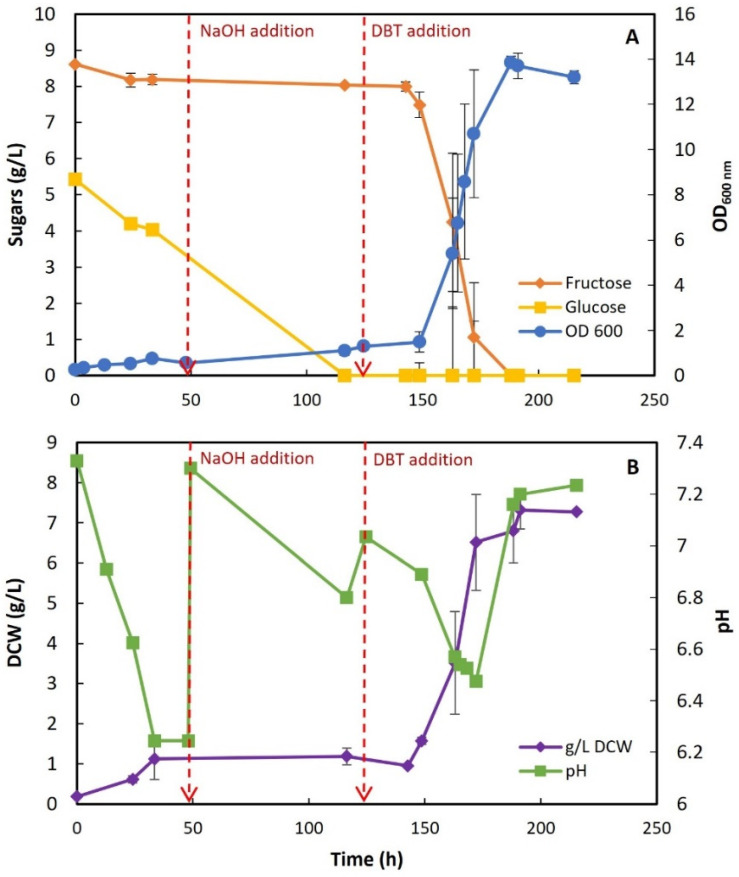
Time-course profiles for cellular growth, sugar consumption, and pH of Gordonia alkanivorans strain 1B cultivated in shake flasks with STFr-liquor containing 14 g/L of reducing sugars at 30 °C and 150 rpm. (**A**)—OD_600_ and sugar (fru/gluc) consumption; (**B**)—DCW and pH variation. Red lines in plots (**A**,**B**) illustrate the time of NaOH/DBT addition. Standard deviation (n = 2) is represented by the error bars.

Using the STFr-liquor from lot #1, a preliminary growth assay (in duplicate) was prepared to evaluate its potential as an alternative C-source and S-source to grow *G. alkanivorans* strain 1B at 30 °C and 150 rpm. Figure 1 represents the time-course profiles for cellular growth and sugar consumption (Figure 1A), as well as the DCW and pH variation (Figure 1B) in strain 1B in shake-flask cultures with the STFr-liquor with 14 g/L of initial total reducing sugars as the C-source. These results show that there was a small initial growth, indicating that there was a residual concentration of sulfur present in the complex liquor that supported the growth. This growth stopped within about 33 h, attaining a maximum OD_600_ of 0.76, with a consumption of mostly glucose (1.4 g/L) and a small amount of fructose (0.5 g/L). This is contrary to the expected result, since strain 1B is described as a fructophilic bacterium [28].

In a previous work, Silva [46] observed that the pH dropped abruptly when an STF-liquor (i.e., a liquor obtained directly from whole-fruit crushing) was used as a C-source for strain 1B growth. So, to avoid the same phenomenon herein, the pH was controlled during the bacterial culture growth. Indeed, within the first 48 h, a significant decrease in pH was observed (from 7.33 to 6.25), and therefore, at 49 h, sterile NaOH was added to readjust the culture pH to 7.30. After this adjustment (Figure 1B), the culture grew slightly, reaching an OD_600_ of 1.1, but only with a residual increase in biomass, accompanied by the complete consumption of the glucose in the liquor (consumption rate: 0.046 g/L/h), but without any fructose consumption up to about 116 h. Overall, these results indicate that some nutrients, such as sulfur, were limiting biomass formation even after the pH adjustment to the optimum value at 49 h.

Since *G. alkanivorans* strain 1B is a bacterium isolated from soil [6], its behavior in the presence of STFr-liquor might be similar to the mechanism described by Hoberg et al. [47] for nutrient extraction from the soil, i.e., through organic acid production and medium acidification. Behavioral alterations, such as preferential glucose consumption instead of fructose, can also be justified by the fact that STFr-liquor is a complex C-source with metal ions that can affect metabolic pathways [34,48].

Because growth was almost nonexistent after 116 h, mainly due to the S-source limitation, DBT was added (to a final concentration of 400 µM in the culture medium) at about 125 h, and the growth was restarted. In these conditions, strain 1B was able to grow, fully consuming the remaining fructose (the only remaining sugar) (Figure 1A) and producing biomass (Figure 1B). Table 2 summarizes the metabolic parameters determined for the culture after DBT addition.

In fact, the DBT pulse did not make an immediate impact, most likely because the cells had to adapt to the new conditions, since the enzymatic “machinery” had to change from sulfate to DBT as the S-source. Thus, after the DBT pulse, a lag phase of about 24 h was observed (until 149 h) and then the bacterium culture entered an exponential phase and stopped growth within about 66 h, reaching a maximum OD_600_ of 13.86 (≈188 h) and a maximum biomass concentration of 7.28 g/L (≈215 h). During the exponential phase, fructose was fully consumed at a maximum rate of 0.27 g/L/h, and strain 1B achieved a µ_max_ of 0.091 h^−1^ with a maximum biomass production rate of 0.236 g_(DCW)_/L/h.

Comparing these metabolic parameters with others reported by previous works with *G. alkanivorans* strain 1B [28,46], it can be observed that the µ_max_ obtained with this STFr-liquor is higher than that obtained with Silva’s STF-liquor (µ_max_ = 0.0523 h^−1^) [46], and similar to the one obtained with a glu–fru (50:50) solution (µ_max_ = 0.091 h^−1^) [28]. Regarding other parameters, a higher biomass production rate was also found (2.2 times higher than that observed by Alves and Paixão [28] using glu–fru (50:50) solution as the C-source), which can be explained by the additional components probably present in the STFr-liquor, which is a complex C-source.

Compared to the STF-liquor studied by Silva [46], this STFr-liquor has much greater stability in terms of pH, with smaller variations over time. In addition, it also has the advantage of presenting a low sulfur content. Indeed, alternative C-sources with excess sulfur, such as Jerusalem artichoke [22] or paper sludge [29], inhibit desulfurization and, thus, require a pretreatment with BaCl_2_ for sulfate removal before their use in BDS assays.

#### 3.2.2. STFr-Liquor Detoxification

Since, after DBT addition (at 125 h), a significant lag phase (~24 h) was observed in the restart of growth (Figure 1A), detoxification assays were also performed to evaluate the possible inhibitory effects of STFr-liquor on strain 1B growth.

In this context, to remove possible growth inhibitors, the STFr-liquor (lot #1) was pretreated with activated charcoal (AC) powder. Different concentrations of AC (1 to 4%) were tested to select the minimum quantity that allows adequate detoxification. Table 3 summarizes the characterization of the different detoxified STFr-liquors obtained.

Different percentages of AC led to different results in terms of color. With lower percentages of AC (1 and 2%), the resultant liquors presented more color, becoming translucid when the AC amount increased (≥3%), indicating a reduction in possible contaminants (e.g., phenolics). In addition, from Table 3, it can be stated that, despite some variation, sugar concentration is maintained in all treatments, but the highest phenolics removal was attained with AC ≥ 3%, changing from 3.72 g/L (untreated liquor) to about 0.2 g/L.

**Figure 2:**
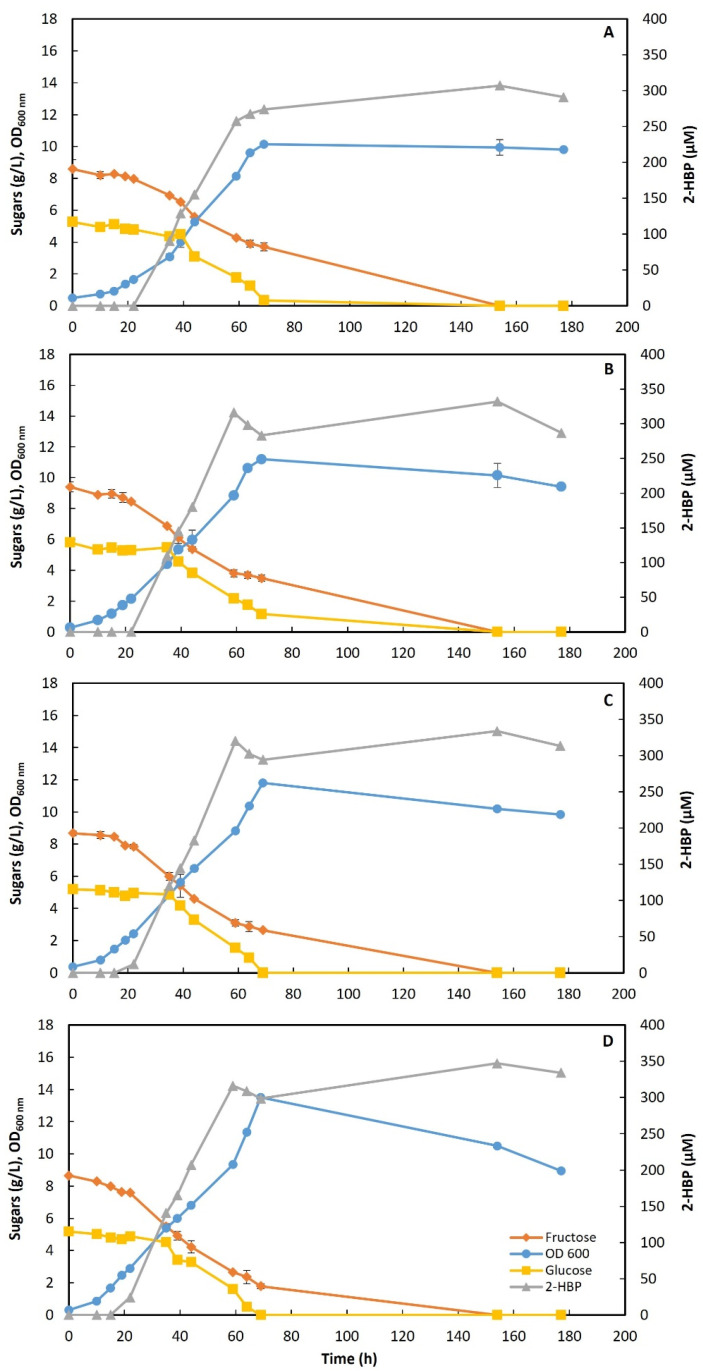
Time-course profiles for cellular growth, sugar consumption, and desulfurization by *G. alkanivorans* strain 1B in shake-flask cultures with detoxified STFr-liquor with activated charcoal: (**A**)—1% AC; (**B**)—2% AC; (**C**)—3% AC; and (**D**)—4% AC; adjusted to an initial concentration of about 14 g/L of reducing sugars and supplemented with 400 µM DBT. Standard deviation (n = 3) is represented by the error bars (SD ≤ 2.62%).

These four detoxified liquors were then tested as C-sources to cultivate strain 1B in shake flasks for DBT desulfurization. These assays were performed in triplicate, with an initial sugar concentration of about 14 g/L total reducing sugars and 400 µM of DBT as the S-source. The results obtained in these BDS assays are illustrated in Figure 2**,** and the corresponding metabolic parameters are described in Table 4.

Strain 1B was able to grow on every condition tested, completely consuming both sugars (fru/glu) and desulfurizing the DBT, regardless of the pretreatment (% AC) used to treat the STFr-liquor. However, as shown in Figure 2 and Table 4, there are differences between results. The lag phase decreased with the detoxification process (15 h for 1% AC; 10 h for ≥2% AC). Then, the exponential phase was maintained up to 69 h for all conditions after the experiment started, reaching a maximum OD_600_ of 10.15 for detox liquor-1; 11.2 for detox liquor-2; 11.8 for detox liquor-3; and 13.5 for detox liquor-4. Similarly, maximum growth rates (µ_max_) also increased with increases in % AC used in the pretreatment (0.086 h^−1^, 0.097 h^−1^, 0.105 h^−1^, and 0.119 h^−1^), as well as the consumption rates of total sugars (0.142 g/L/h, 0.153 g/L/h, 0.162, and 0.175 g/L/h, for detox liquor-1 to -4, respectively).

At 177 h, all growth in the detox liquors attained maximum biomass concentration, namely 4.55 g/L, 4.52 g/L, 4.37 g/L, and 4.43 g/L for detox liquor-1, -2, -3, and -4, respectively. Moreover, both sugars (fru/glu) were fully consumed in all conditions within 154 h, despite the consumption profile still not following the true fructophilic behavior usually observed with this strain. In fact, the detoxified STFr-liquor added as the C-source, to the culture medium, at an initial concentration of 14 g/L of total sugars still presented some inhibition of bacterial fructophily.

Regarding biodesulfurization, the initial 2-HBP production was detected at 22 h for detox liquors-3 and -4 (11.72 µM and 24.30 µM, respectively), and only at 35 h for detox liquors-1 and -2 (90.7 µM and 109.4 µM, respectively). Furthermore, the maximum 2-HBP value, attained at 154 h, increased with increases in % AC used in the pretreatment: 307 µM, 332 µM, 334 µM, and 347 µM for detox liquors-1 to -4 (Figure 2, Table 4). Most probably, these maximum 2-HBP values are undervalued because of missing earlier sampling. Moreover, the corresponding maximum production rates were 7.19 µM/h, 8.60 µM/h, 8.88 µM/h, and 8.83 µM/h for detox liquors-1, -2, -3 and -4, respectively. Although the maximum value for the 2-HBP production rate was observed for detox liquor-3 (8.88 µM/h), the values obtained for detox liquor-3 and -4 were very similar.

Overall, comparing the different metabolic parameters (Table 4) for the four detoxified STFr-liquors, the best pretreatment seems to be the detoxification using 4% AC. Indeed, when the liquor-4 was used as the C-source, the best results, in terms of the key parameters for BDS, were observed: (1) the highest growth (OD_600_ = 13.5); (2) the highest growth rate (µ_max_ = 0.119 h^−1^); (3) the highest sugar consumption rate (0.175 g/L/h); and (4) the highest 2-HPB production (347 µM). So, the detoxification process using 4% AC was selected as the best methodology to treat the STFr-liquor in further assays.

### 3.3. Chemostat Assays

Before the scale-up of a biotechnological process, it is necessary to obtain a better understanding of the cellular behavior and physiology of the microorganism responsible for the bioprocess in order to increase process efficiency and reduce expenses. This is especially important for the BDS process, to which the overall costs are highly dependent on bacterial biocatalyst production [7].

In this context, based on the results obtained previously in the shake-flask assays, the next step was to optimize the culture conditions to use STFr-liquor as a potential alternative low-cost C-source for biocatalyst production in chemostats (continuous culture).

#### 3.3.1. Preliminary Assays

*G. alkanivorans* strain 1B has previously been cultivated in chemostats; however, most results were obtained with either glucose or fructose [10,49]. Given the complexity of STFr-liquor as a C-source, it was considered fundamental to first establish baseline results using the culture medium supplemented with pure commercial sugars in the same ratio present in the liquor.

Therefore, the first set of assays was carried out using a mix of commercial fructose and glucose (Fru + Glu) in 6:4 ratio, respectively. Culture conditions were maintained as previously established [10,11] (i.e., a 1 L vase with 435 mL SFMM medium supplemented with 10 g/L of total sugars, at 30 °C, pH 7.5, 410 rpm agitation, 2 vvm aeration rate, and 0.05 h^−1^ dilution rate) for five turnovers of the culture medium, after which it was considered that the steady state was achieved.

Under these conditions, the culture of *G. alkanivorans* strain 1B reached an OD_600_ of 4.45, but only consumed half of the sugars present in culture medium (with about 5 g/L of total sugars remaining: 3.2 g/L glu and 1.8 g/L fru). These values were clearly lower than what was observed during the shake-flask assay and contrasted with previous results obtained with fructose, in which 10 g/L of sugar was consumed, leading to the production of approximately 5 g/L of bacterial biomass [10].

Considering that this strain typically consumes glucose at lower rates [28], it was supposed that the dilution rate could be preventing the cells from fully consuming the sugar mix. As such, maintaining the remaining conditions, the dilution rate was lowered to 0.04 h^−1^. This reduction resulted in an increase in OD_600_ to 9.8, as well as an increase in carbon consumption to approximately 8 g/L; nevertheless, there was still 2 g/L of total sugars not consumed (1.6 g/L glu and 0.4 g/L fru). Further reductions in dilution rates were tested (0.04–0.02 h^−1^), but complete sugar consumption was never observed.

In an attempt to observe complete sugar consumption, and given that 8 g/L (of fructose and glucose mix) was being consumed at 0.04 h^−1^, a different culture medium formulation was tested. The concentrations of all medium components were reduced to half, thus lowering sugar concentration to 5 g/L, below the 8 g/L threshold. However, even under these conditions, sugars were not fully consumed. This clearly indicated that the issue was no longer related to the dilution rate, but instead pointed towards a nutritional deficiency.

In previous works [13,48], it was demonstrated that, using the strain 1B, glucose consumption and biomass production were greatly affected by the presence and concentration of some medium components, namely components of trace element solutions. Since the composition of the SFMM medium was optimized/minimized, using fructose as the main C-source [8], it was hypothesized that some nutrient limitations could be influencing the behavior of the strain. So, a new chemostat assay was settled using the same culture medium as in the shake-flask assays, i.e., the SFM medium. This culture medium was tested with about 5 g/L C-source (Fru + Glu) at a dilution rate of 0.04 h^−1^. In these conditions, complete sugar consumption was attained. The main metabolic parameters for this #SS-culture (Fru + Glu) are described in Table 5. These results indicate that there was a nutritional limitation when the SFMM medium was used, which was previously undetected when Pacheco et al. [8] used ≥80% fructose as the C-source, resulting in a slower growth rate/sugar consumption.

#### 3.3.2. Assays with STFr-Liquor

Having established the best culture conditions for Fru + Glu mix (6:4 ratio) as a C-source, a second set of assays was carried out with STFr-liquor (lot #2, Table 1) and the corresponding liquor after the detoxification with 4% AC (detox STFr-liquor) as alternative C-sources. However, the use of complex C-sources, like those from agro-industrial residues, presents an added advantage of supplementation with extra nutrients, which could suppress the above-referred limitation. As such, these assays were performed using the SFMM culture medium supplemented with each STFr-liquor (set to an initial concentration of ~5 g/L of Fru + Glu).

The first assay performed using STFr-liquor as the C-source replicated the conditions successfully tested with the Fru + Glu mix. STFr-liquor was added to the culture medium to obtain 5.4 g/L reducing sugars, and the dilution rate was maintained at 0.04 h^−1^ (STFr L1). This resulted in the complete consumption of available sugars, leading the production of 2.43 g/L of biomass (Table 5).

The next approach, with the same STFr-liquor (for initial 5.4 g/L reducing sugars), was to increase the dilution rate to 0.05 h^−1^ (STFr L2) to generate cells with greater metabolic activity. Although an overall increase in metabolic activity was observed, this increase in dilution rate resulted in an accumulation of 1 g/L of the carbon source, reinforcing the notion that the glucose present in liquor inhibits greater dilutions rates using the strain 1B.

Given these results, a final assay was performed using the detox liquor (for initial 4.72 g/L reducing sugars) at a dilution rate of 0.04 h^−1^ (DTX STFr L). This resulted in the complete consumption of sugars in the medium, with the production of 2.40 g/L of biomass, similar to the untreated liquor. The main metabolic parameters for the #SS-cultures of these assays are summarized in Table 5.

Since the operational conditions were maintained at a constant level, the behavior of the four #SS-cultures is related to the physiological response of the bacterial cells to the influence of the C-source on the culture medium used (SFM/SFMM).

Comparing all #SS-cultures, the lowest biomass production was observed in Fru + Glu (1.89 g/L), while the highest biomass concentration was achieved in STFr L2 (2.79 g/L), despite using a less concentrated culture medium (SFMM) and a faster dilution rate (0.05 h^−1^). Using the STFr-liquor, regardless of the condition tested, even with SFMM medium, there were always enough nutrients to generate more biomass than when using commercial sugars with a non-minimized culture medium (SFM), possibly due to STFr-liquor being a complex C-source with additional nutrients. Comparing the two #SS-cultures obtained with STFr-liquor (STFr L1 vs. STFr L2), it can be observed that the increase in dilution rate (0.04 to 0.05 h^−1^) seems to channel the metabolism towards the production of biomass, since with less total sugar consumption there was more biomass produced, as indicated by the carbon conversion efficiency (CCE), which indicates the percentage of consumed carbon that is used in the cellular biomass. In DTX STFr L, grown with less initial sugars (4.72 g/L) and at 0.04 h^−1^, there was almost the same biomass being produced as in STFr L1 (≈2.4 g/L DCW for both cultures), once again with a high amount of carbon being used in the cell constitution.

#### 3.3.3. BDS Assays Using Resting Cells

To correctly evaluate desulfurization activity in a system in which biocatalyst production and biodesulfurization occur separately, the conversion of DBT to 2-HBP was analyzed through resting cell assays (see Section 2.6). Unlike growing cells, these assays occur in the absence of nutrients or C-sources, preventing changes in cell metabolism or cell proliferation. This allows the testing of BDS ability under constant reproducible conditions with a fixed cell concentration.

Comparing the results obtained in the different sets of chemostat assays (Table 5), the highest 2-HBP specific production rate (*q*_2-HBP_) was achieved with cells from DTX STFr L (6.98 µmol/g_(DCW)_/h). However, the maximum 2-HBP production (212.68 µM) was observed with cells from STFr L1, which finished BDS 3.5 h earlier than DTX STFr L cells, presenting a *q*_2-HBP_ of 6.50 µmol/g_(DCW)_/h. In contrast, the cells from STFr L2 presented the lowest 2-HBP production (51.87 µM) and the lowest corresponding *q*_2-HBP_ (1.84 µmol/g_(DCW)_/h), since the dilution rate was too high, leading to the accumulation of nutrients, sulfates, and C-sources.

Comparing the *q*_2-HBP_ values from the four #SS-cultures, it is possible to state that both STFr-liquor and detoxified STFr-liquor produced biocatalysts with higher desulfurization ability than the commercial sugar mix (Fru + Glu) when used as a C-source at a dilution rate of 0.04 h^−1^. Indeed, *q*_2-HBP_ values obtained with cells from STFr L1 (6.50 µmol/g_(DCW)_/h) or cells from DTX STFr L (6.98 µmol/g_(DCW)_/h) were much higher than the *q*_2-HBP_ value obtained with cells from Fru + Glu (4.32 µmol/g_(DCW)_/h). Contrary to commercial sugars, complex C-sources, such as STFr-liquor, may have supplementary nutrients that can support the highest biomass production (>2.4 g/L) observed in STFr L1 and DTX STFr L cultures (Table 5). In Fru + Glu #SS-culture, the maximum biomass production attained was 1.89 g/L.

Comparing these results to the ones from the shake-flask assays, the highest *q*_2-HBP_ was also obtained when detoxified STFr-liquor was used (*q*_2-HBP_ = 5.06 µmol/g_(DCW)_/h for shake-flask assay). However, the *q*_2-HBP_ results from both the STFr L1 and DTX STFr L chemostat cultures were greater than those observed for all shake-flask cultures, showing that the use of resting cells grown in controlled conditions is beneficial to increase BDS activity. Moreover, contrary to what was observed in the shake-flask assays, in the chemostat cultures, no growth inhibition was observed due to the STFr-liquor. So, no prior detoxification process is needed. This greatly facilitates its application at a larger scale, since it simplifies the process needed to obtain a fermentable liquor.

Overall, these results indicate that it could be possible to use an agro-industrial residue as a sustainable alternative C-source to produce cost-effective biocatalysts with high desulfurization ability. Furthermore, the analysis of the metabolic parameters obtained for the different #SS-cultures (Table 5) points out that the culture conditions used in the STFr L1 culture (STFr-liquor as C-source at a dilution rate of 0.04 h^−1^) are the best conditions for further scale-up studies towards an industrial process.

Several works describe the use of different C-sources as alternatives to conventional sugars for the cultivation of strain 1B cells for BDS. Table 6 lists the different C-sources and pretreatments tested, as well as the sugar concentration and resulting BDS activity of strain 1B when cultivated with each of them.

As mentioned throughout the work and previously demonstrated [28], *Gordonia alkanivorans* strain 1B is a fructophilic bacterium. As such, conventional agricultural waste streams rich in starch, sucrose, or cellulose, which mostly generate glucose-rich syrups, are not ideal, since they lead to lower metabolic and desulfurization rates. This is evidenced by the results obtained with recycled paper sludge hydrolysate, which are the lowest in terms of BDS activity (1.1 µmol/g_(DCW)_/h, Table 6), as well as the results using sugar beet molasses, which present 50% glucose concentration, but still resulted in the third lowest BDS activity (3.12 µmol/g_(DCW)_/h, Table 6).

Comparing the different results in Table 6, it is possible to observe that, despite presenting the second-lowest concentration of sugars, STFr-liquor allowed for the second-highest BDS activity (6.98 µmol/g_(DCW)_/h) for this strain, only surpassed by Jerusalem artichoke juice (JAJ) after enzymatic hydrolysis and sulfate precipitation (8.33 µmol/g_(DCW)_/h). Furthermore, unlike JAJ and others, STFr-liquor is not dependent on hydrolysis, and does not require detoxification nor initial sulfur removal (by dialysis or BaCl_2_ precipitation), since its sulfur content is sufficiently low to avoid the inhibition of BDS activity. Moreover, it was successfully used as both a carbon and partial nutrient source, contributing to reducing overall nutrient demands, and allowed the maintenance of several stable continuous cultures for several days, indicating that it could be used for prolonged cultivation periods.

The two greatest bottlenecks for using STFr-liquor as a C-source are clearly the variability in sugar concentration and the seasonality of the fruits. These are bottlenecks shared by many waste-based feedstocks and are consequences of using agro-industrial residues, which limits the amount of control over quality and the availability of the alternative C-source.

However, despite the variability in global sugar concentration, it is important to point out that the fructose/glucose ratio was mostly maintained, which, as discussed throughout this work, is fundamental for the cultivation of strain 1B.

Moreover, despite the seasonality of the fruits (STFs), which are mostly available during the autumn and early winter, STFr-liquor is produced from residues of the production of jam, which can occur throughout the year, as fruits can be frozen for subsequent processing. Nevertheless, STFr-liquor should be considered not as a single feedstock to be used all year, but instead as one of several other alternatives that can be used in a biorefinery according to their moment-to-moment availability.

### 3.4. Production of Added-Value Bioproducts

As already referenced, to obtain a scalable BDS process with strain 1B as the desulfurizing microorganism, while also producing effective biocatalysts using low-cost alternative C-sources to reduce microbial production costs (such as STFr-liquors), it is also necessary to explore possible high-added-value byproducts to create sustainable and economically viable biorefinery. Indeed, to profit from the overall bioprocess, it is important to retrieve all the value from microbial biomass as an integrated approach.

#### 3.4.1. Carotenoids

Total carotenoid production was evaluated for the different biomasses from the three #SS-cultures obtained in the chemostat assays at a dilution rate of 0.04 h^−1^: STFr L1, DTX STFr L, and Fru + Glu. Carotenoids were first extracted from each biomass using the ionic liquid + ethyl acetate mix method described by Silva et al. [43], and then quantified through spectrophotometry analysis (Table 7). The highest production value was obtained by the culture grown with untreated STFr-liquor, where total carotenoids reached 338 ± 15 μg/g_(DCW)_ (STFr L1), followed by the culture with commercial sugars with 125 ± 0.3 μg/g_(DCW)_ (Fru + Glu). The lowest carotenoid production, with only 106 ± 2 μg/g_(DCW)_, was observed when detox STFr-liquor was used as the C-source (DTX STFr L).

These values of total carotenoids produced, without any light control, are similar or higher than the values described by Pacheco [50] for cultures of *G. alkanivorans* strain 1B grown in a chemostat with 10 g/L of C-source, where fructose was the predominant sugar (Fru + Glu (8 + 2 g/L); JAJ—Jerusalem artichoke juice): Fru + Glu ⇒ 134 μg/g_(DCW)_; JAJ ⇒ 121 μg/g_(DCW)_. Moreover, in the current study, the highest value of carotenoids extracted from the biomass of strain 1B (STFr L1: 338 μg/g_(DCW)_) is 49% higher than the value described for *G. jacobaea* (CECT 5282), a strain of genetically modified *Gordonia* used to produce pigments (227 μg/g_(DCW)_) [31,51,52,53].

These are very promising results for obtaining microbial pigments using a sustainable alternative C-source (STFr-liquor), especially since conditions were not optimized considering the key parameters for carotenoid induction. Thus, further optimization can be performed to achieve viable industrial application (e.g., in the agro-food industry).

#### 3.4.2. Gordofactin BS/BE

Other potential added-value bioproducts to consider in the BDS process by strain 1B are surface-active biomolecules. Indeed, during its growth, strain 1B is able to produce gordofactin [18], a BS/BE that is released into the culture broth.

Thus, in addition to the production of carotenoids within the biomass, the production and release of gordofactin to the culture broth from different #SS-cultures in chemostat assays was also evaluated. It is difficult to correctly assess surfactant/emulsifier production, especially when the molecule structure is unknown. Therefore, it is important to have a comparative method to quantify the presence of these types of compounds. In this context, emulsifying activity (EA, U/mL) was evaluated in the cell-free supernatant of each #SS-culture after centrifugation (10,000× *g*, 10 min), using the method developed by Tavares et al. [16]. This new method defines the emulsification unit as the minimum volume of product (Vol_min_ of BS/BE, up to 1 mL) required to form and maintain 100% emulsion in the organic phase (1 mL of n-heptane).

Table 7 shows the results of EA (U/mL) for the three #SS-cultures from the chemostat assays performed at a dilution rate of 0.04 h^−1^. The highest emulsifying activities were achieved in the following #SS-cultures: STFr L1 (EA = 8 U/mL) and DTX STFr L (EA = 7.69 U/mL), with 5.4 and 4.72 g/L of sugars consumed, respectively. These results are especially interesting, since they are slightly higher than those previously reported by Tavares et al. [16] (EA ≈ 7.3 U/mL) using the same bacterium, but cultivated in shake flasks with a much greater initial concentration of the C-source (10 g/L fructose).

Gordofactin was previously demonstrated to present an emulsifying activity in line with several industrial and domestic detergents, even surpassing some commercial brands [16]. Furthermore, it can act on a broad range of hydrophobic liquids; it is remarkably stable across a broad range of temperatures (30 °C to 80 °C), pH values (pH 3–12), and salt concentrations (1 to 100 g/L NaCl) [18]. Lastly, it also shows some antioxidant and antibiotic activity [18]. These characteristics give it increased biotechnological potential and open prospects for further gordofactin valorization, contributing to the global economic viability of a BDS process based on a biorefinery using *G. alkanivorans* strain 1B.

Additionally, net-zero CO_2_ emissions could be achieved if the heterotrophic bioreactor (*G. alkanivorans* strain 1B) is coupled with an autotrophic bioreactor (e.g., *Haematococcus pluvialis* microalga), as described by Tavares et al. [54], which may also produce additional added-value bioproducts (e.g., astaxanthin) that can contribute to lowering the overall biorefinery costs.

## 4. Conclusions

The main goal of this work was to evaluate the potential for the valorization of an agro-industrial residue, STFr, as a sustainable alternative C-source to obtain cost-effective biocatalysts and additional high-market-value bioproducts in a fuel BDS refinery using *G. alkanivorans* strain 1B.

In this context, a STFr-liquor rich in fructose (60% fructose/40% glucose) was prepared and tested in shake flasks to evaluate both the growth and desulfurization ability of strain 1B using this alternative C-source. This initial set of assays indicated an inhibitory effect from STFr-liquor, which was successfully eliminated by treating the liquor with activated charcoal (AC). Indeed, the best growth and BDS results were obtained when STFr-liquor treated with 4% AC was used as the C-source.

After identifying the metabolic behavior of the bacterium in shake flasks for the alternative C-sources (i.e., treated/untreated STFr-liquors) vs. the commercial C-source (fru/glu mix, 6:4 ratio), further optimization assays were carried out using a chemostat (1L bioreactor) to obtain #SS-cultures. The cells produced in the bioreactor demonstrated significant BDS activity, especially cells obtained from the #SS-cultures STFr L1 (6.50 µmol/g_(DCW)_/h) and DTX STFR L (6.98 µmol/g_(DCW)_/h). Indeed, the DBT desulfurization results demonstrated that both STFr-liquor and detoxified STFr-liquor produced biocatalysts with higher desulfurization ability than commercial sugars (Fru + Glu) when used as a C-source at a dilution rate of 0.04 h^−1^. Furthermore, the use of STFr-liquor as a C-source allowed the utilization of a sulfur-free mineral-minimized (SFMM) culture medium with reduced added nutrient concentration, without compromising (and even increasing) biomass production, possibly due to the additional nutrients in its complex composition.

To conclude, the overall metabolic results from the chemostat assays point to the use of untreated STFr-liquor as a sustainable feedstock to devise cost-reduction strategies for the production of cheaper biocatalysts with high desulfurization ability. Moreover, both the carotenoid and gordofactin results (338 ± 15 µg/g_(DCW)_ and EA = 8 U/mL, respectively) open prospects for further bioproduct valorization, contributing to the global economic viability of a BDS process with *G. alkanivorans* strain 1B biorefinery as an integrative process in order to achieve BDS scale-up.

## Figures and Tables

**Table 1:** Characterization of STFr-liquors.

STFr-Liquor Lot (#)	Fructose (g/L)	Glucose (g/L)	Total Sugars (g/L)	Phenolics (g/L)
1	66.22	43.40	109.62	3.72
2	41.35	24.65	66.00	4.12
3	63.72	43.38	107.10	3.15

Note: The results are the mean value of three replicates (SD ≤ 4.2 ± 0.5%, SD = standard deviation).

**Table 2:** Metabolic parameters for the growth of *G. alkanivorans* strain 1B with STFr-liquor (lot #1) containing about 8 g/L fructose after DBT addition (400 µM).

Metabolic Parameters	
Maximum OD_600 nm_	13.86
Maximum growth rate (h^−1^)	0.091
Maximum biomass concentration (g/L)	7.28
Maximum fructose consumption rate (g/L/h)	0.270
Maximum biomass production rate (g_(DCW)_/L/h)	0.236

**Table 3:** Characterization of detoxified STFr-liquors in terms of fructose/glucose, total sugars, and total phenolics.

AC (%)	Detoxified STFr-Liquor			
	Fructose (g/L)	Glucose (g/L)	Total Sugars (g/L)	Phenolics (g/L)
1%	60.09	36.86	96.96	1.02
2%	65.80	40.61	106.41	0.32
3%	60.72	36.31	97.03	0.16
4%	60.59	36.32	96.90	0.21

Note: The results are the mean value of three replicates (SD ≤ 5.0 ± 0.5%).

**Table 4:** Metabolic parameters for DBT desulfurization by *G. alkanivorans* strain 1B in shake-flask cultures with detoxified STFr-liquor (14 g/L total reducing sugars) as C-source.

Metabolic Parameters	Detoxified STFr-Liquor			
	1% AC ^1^	2% AC ^2^	3% AC ^3^	4% AC ^4^
Maximum OD_600nm_	10.15	11.20	11.80	13.50
Maximum growth rate (h^−1^)	0.086	0.097	0.105	0.119
Total sugars consumption rate (g/L/h)	0.142	0.153	0.162	0.175
Maximum biomass (g/L)	4.55	4.52	4.37	4.43
Maximum 2-HBP production rate (µM/h)	7.19	8.60	8.88	8.83
Maximum 2-HBP produced (µM)	307	332	334	347

^1^ Detox liquor-1; ^2^ detox liquor-2; ^3^ detox liquor-3; ^4^ detox liquor-4. SD ≤ 5.0 ± 0.5%.

**Table 5:** Metabolic parameters of the #SS-cultures of *G. alkanivorans* strain 1B grown in chemostat in SFM culture medium with STFr-liquor (lot #3; STFr L1 and STFr L2—5.4 g/L total sugars) or detox STFr-liquor (STFr-liquor treated with 4% AC; DTX STFr L—4.72 g/L total sugars) as C-source and in SFM culture medium with commercial sugars (Fru + Glu—5.4 g/L total sugars) as C-source.

	SFM Culture Medium	SFMM Culture Medium with STFr-Liquor		
	Fru + Glu	STFr L1	STFr L2	DTX STFr L
Dilution rate (h^−1^)	0.04	0.04	0.05	0.04
Initial carbon (g/L)	5.4	5.4	5.4	4.72
Initial sulfate (mg/L)	10.14	10.14	10.14	10.14
Accumulated C-source (g/L)	0.11	0	1.0	0
OD_600nm_	5.34 ± 0.3	6.88 ± 0.1	6.75 ± 0.1	6.72 ± 0.08
Biomass (g/L)	1.89 ± 0.13	2.43 ± 0.04	2.79 ± 0.07	2.40 ± 0.07
Biomass production rate (g/L/h)	0.076	0.097	0.140	0.096
C-source consumption rate (g/L/h)	0.212	0.216	0.220	0.191
*q*_C-source_ (mmol/g_(DCW)_/h)	0.62 ± 0.04	0.49 ± 0.01	0.44 ± 0.01	0.38 ± 0.01
*q*_CO2_ (mmol/g_(DCW)_/h)	0.42 ± 0.09	0.20 ± 0.05	0.31 ± 0.007	0.35 ± 0.01
*q*_O2_ (mmol/g_(DCW)_/h)	4.93 ± 1.04	3.50 ± 0.07	2.29 ± 0.05	2.66 ± 0.07
CR (%)	43.29 ± 1.7	47.46 ± 1.4	68.69 ± 1.34	58.62 ± 1.28
CCE (%)	32.21 ± 2.3	40.56 ± 0.8	57.07 ± 1.34	45.25 ± 1.28
Maximum 2-HBP (µM)	199.69 ± 4.44	212.68 ± 1.55	51.87 ± 1.44	66.97 ± 2.15
*q*_2-HBP_ (µmol/g_(DCW)_/h)	4.32 ± 0.03	6.50 ± 0.14	1.84 ± 0.11	6.98 ± 0.04

*q*_C-source_—specific rate of C-source (Fru + Glu) consumption; *q*_CO2_—specific rate of carbon dioxide production; CR—carbon recovery (percentage of consumed carbon that is used in the cell constitution); CCE—carbon conversion efficiency; *q*_2-HBP_—specific rate of 2-HBP production.

**Table 6:** Alternative carbon sources tested to cultivate *G. alkanivorans* strain 1B for biodesulfurization assays.

Carbon Source	Sugars (g/L)	Pretreatment	BDS Activity (µmol/g_(DCW)_/h)	References
Recycled paper sludge hydrolysate	53.9	Enzymatic hydrolysis and dialysis	1.1	[29]
Carob pulp liquor	300	Water extraction, acidic hydrolysis and sulfate precipitation	1.56	[19,46]
Jerusalem artichoke juice	145	Acidic hydrolysis and sulfate precipitation/enzymatic hydrolysis and sulfate precipitation	5.06/8.33	[22,23]
Strawberry tree fruit liquor	173	Direct extraction	5.0	[46]
Sugar beet molasses	500	Acidic hydrolysis and sulfate precipitation	3.12	[21]
Strawberry tree fruit residue liquor	109.62/ 66.0	Untreated/AC treatment	6.50/6.98	Present work

**Table 7:** Carotenoid and gordofactin production by *G. alkanivorans* strain 1B on the three #SS-cultures grown in chemostat at a dilution rate of 0.04 h^−1^: STFr L1; DTX STFr L; and Fru + Glu.

	SFM Culture Medium	SFMM Culture Medium with STFr-Liquor	
	Fru + Glu	STFr L1	DTX STFr L
Total carotenoids (μg/g_(DCW)_)	125.0 ± 0.3	338.0 ± 15.0	106.0 ± 2.0
EA (U/mL)	6.25	8.0	7.69

## Data Availability

The authors confirm that the datasets supporting the findings and conclusions of this study are available within the article.

## Supplementary Materials

The following supporting information can be downloaded at: https://www.mdpi.com/article/10.3390/molecules30102137/s1. Figure S1—Scheme for liquor preparation from STF residue.
