# Supplementary figures and images for: Strawberry Tree Fruit Residue as Carbon Source Towards Sustainable Fuel Biodesulfurization by Gordonia alkanivorans Strain 1B
Source: Molecules. 2025 May 13;30(10):2137. doi: 10.3390/molecules30102137 (PMC12114536; doi:10.3390/molecules30102137)

## Supplementary Material

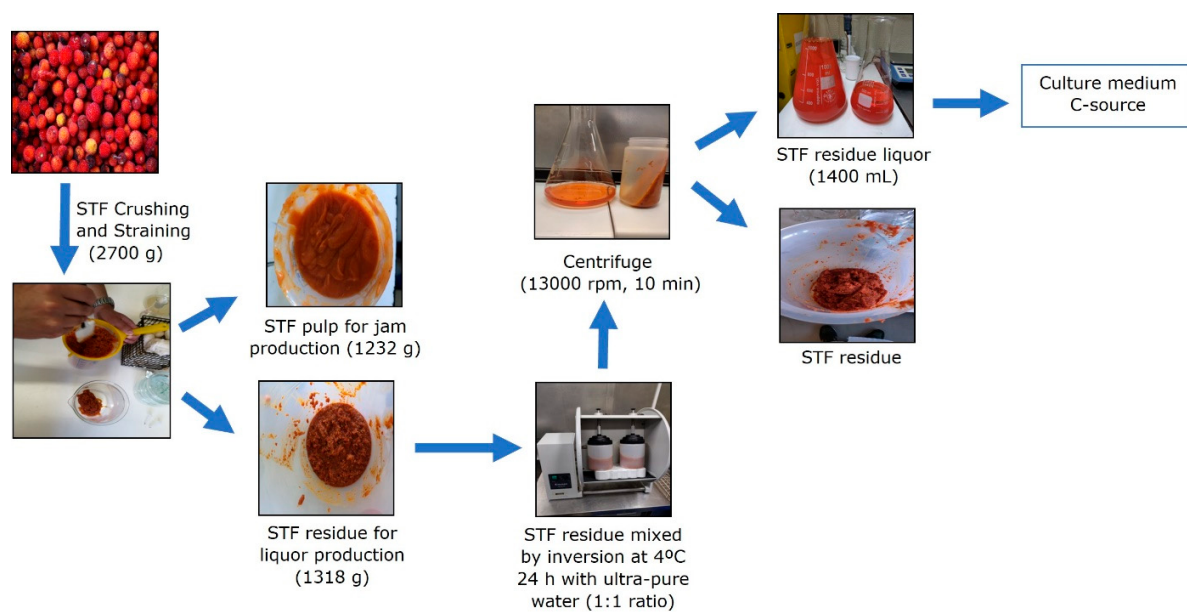

**Figure S1.** Scheme for liquor preparation from STF residue.

Supplement: Supplementary file 1 [file molecules-30-02137-s001.zip › molecules-3580301-supplementary.pdf]
